# Single nucleotide polymorphisms: Implications in the early diagnosis and targeted intervention of coronary microvascular dysfunction

**DOI:** 10.1016/j.gendis.2024.101249

**Published:** 2024-02-28

**Authors:** Dingyuan Tian, Jie Li, Xiaoyue Lai, Qingyuan Yang, Zhihui Zhang, Fang Deng

**Affiliations:** a Department of Pathophysiology, College of High Altitude Military Medicine, Army Medical University, Chongqing 400038, China; b Department of Cardiovascular Medicine, Southwest Hospital, Army Medical University, Chongqing 400038, China; c Department of Ultrasound, Xinqiao Hospital, Army Medical University, Chongqing 400037, China; d Center for Circadian Metabolism and Cardiovascular Disease, Southwest Hospital, Army Medical University, Chongqing 400038, China; e Key Laboratory of Geriatric Cardiovascular and Cerebrovascular Diseases, Ministry of Education, Chongqing 400038, China; f Key Laboratory of Extreme Environmental Medicine, Ministry of Education of China, Chongqing 400038, China; g Key Laboratory of High Altitude Medicine, PLA, Chongqing 400038, China

**Keywords:** Coronary artery disease, Coronary microvascular dysfunction, Mechanism, Prediction, Single nucleotide polymorphism

## Abstract

Coronary microvascular dysfunction (CMD) is a clinical syndrome of myocardial ischemia caused by structural and/or functional abnormalities of pre-coronary arterioles and arterioles. While genetics and other factors play a role in CMD etiology, the key pathogenic mechanism remains unclear. Currently, the diagnostic procedure for CMD is still cumbersome, and there is a lack of effective targeted interventions. Single nucleotide polymorphisms (SNPs) offer promise in addressing these issues. SNPs, reflecting common genetic variations, have garnered extensive investigation across multiple diseases. Several SNPs associated with CMD have been discovered, and some have the potential to be therapeutic targets. Nevertheless, studies on CMD-related SNPs are relatively nascent and limited in number. In this review, we summarize the previously reported CMD-associated SNPs, delineate their pathophysiological mechanisms, and predict potentially important CMD sites by analyzing the SNPs linked to diseases sharing similar pathogenetic mechanisms and risk factors, such as coronary artery disease. We aim to explore reliable genetic markers implicated in CMD risk and prognosis, thereby providing a novel approach for early diagnosis and gene-targeted interventions of CMD in subsequent studies.

## Introduction

The coronary artery comprises epicardial arteries (diameter >500 μm), pre-arterioles (500 μm > diameter >100 μm), arterioles (100 μm > diameter >10 μm), and capillaries, with pre-arterioles, arterioles, and capillaries collectively constituting the coronary microcirculation.^1^ Coronary microvascular dysfunction (CMD) refers to structural and functional alterations in pre-arterioles and arterioles that can lead to coronary blood flow impairment and ultimately myocardial ischemia.^1^ Approximately 50–70 percent of patients with myocardial ischemia and no obstructive arteries are considered to have concurrent CMD. This proportion reaches as high as 80 percent in the female population, correlating with a poor prognosis.^2^, ^3^, ^4^ Despite this prevalence, the key pathogenetic mechanism of CMD remains largely unknown, and effective targeted interventions are lacking.^5^^,^^6^ Consequently, robust exploration of CMD pathogenesis is crucial for studies aimed at improving diagnosis and treatment.

Genetics plays an important role in the occurrence and development of coronary artery disease (CAD). Recently, detecting risk loci of CAD has become one of the important methods for identifying high-risk patients or providing them with specific therapies.^7^^,^^8^ Single nucleotide polymorphism (SNP) refers to a DNA sequence polymorphism caused by a single nucleotide variation.^9^ The clinical transformation of SNP sites of cardiovascular disease including CAD has progressed rapidly. For example, detecting SNPs in the proprotein convertase subtilisin/kexin type 9 (*PCSK9*) gene is beneficial to diagnosing hypercholesterolemia and determining the risk of atherosclerosis (AS) and CAD.^10^ Similarly, genotyping of the vitamin K epoxide reductase complex 1(VKORC1) can predict a high risk of overdose before initiation of anticoagulation therapy and facilitate the development of a personalized anticoagulation treatment regimens.^11^ Therefore, the search for SNP sites is consequently highly significant for enhanced diagnosis, precise therapies, and improved prognosis of these diseases.

Nevertheless, few SNP studies have focused on CMD, and a large number of CMD-related SNPs have yet been found. In this article, we give an overview of the limited known CMD-associated SNPs from the view of coronary microvascular structure and function, and we predict potential loci that could significantly drive the development of CMD by highlighting SNPs associated with its pathogenesis and risk factors. We aim to provide a novel approach for subsequent SNP-related research on CMD diagnosis and precise prevention and treatment for CMD.

## What are SNPs?

As outlined above, SNPS are variations in DNA sequences that involve the substitution of a single nucleotide base for another within a genome. Typically occurring in SNPs usually occur in non-coding regions, SNPs can influence the structure and function of targeted proteins, especially when they occur at regulatory sites of genes. This can contribute to the occurrence and development of multiple diseases.^9^

Once proposed by Eric S. Lander as the third-generation molecular marker in 1996, SNPs are characterized by their abundant presence throughout the genome, easy automatic analysis, and high genetic stability.^12^ Over recent decades, multiple advances in sequencing technology and the decreasing costs of genetic testing, have facilitated extensive clinical research aimed at identifying disease-associated and disease-causing variants.^13^ To date, SNPs have been widely used in biological and medical research fields such as human disease gene screening, disease diagnosis and risk prediction, and personalized drug screening.^14^^,^^15^ The heritability of risk factors and regulatory function of pleiotropic region genes on cardiovascular disease have been widely acknowledged.^16^ Thus, finding CMD-associated SNP sites are beneficial to improve the diagnosis, precise therapy, and prognosis of CMD.

## The pathogenesis and risk factors of CMD

The pathogenesis of CMD involves structural and/or functional remodeling of the coronary microcirculation due to the dysfunction of endothelial cells (ECs) and vascular smooth muscle cells (VSMCs), as well as microvascular remodeling.^1^ Among them, coronary structural abnormalities mainly include microvascular occlusion and remodeling, while functional abnormalities include the dysfunction of microvascular vasoconstriction and vasodilatation.^17^^,^^18^ However, the mechanisms underlying CMD pathogenesis remain unclear. At present, oxidative stress and the consequent inflammatory response are regarded as the key mechanisms of CMD progression.^19^ Impaired endothelial-dependent vasomotor activity is manifested as impaired nitric oxide (NO)-mediated vasodilation due to intracellular reactive oxygen species overproduction or enhanced endothelin-1 (ET-1)-mediated vasoconstriction through activation of RhoA/Rho-kinase pathway.^20^^,^^21^ RhoA/Rho-kinase has also been implicated in VSMC hypercontraction leading to the spasm of coronary vessels and inflammation in ECs and VSMCs.^21^ Diabetes, hyperlipidemia, and hypertension are the most important risk factors for CMD.^22^, ^23^, ^24^ Several studies have reported that impaired endothelial NO production in the microcirculation due to endothelial dysfunction and vascular insulin resistance, as well as microvascular rarefaction and diminished angiogenesis, could lead to myocardial perfusion defects in patients or animals with diabetes.^25^^,^^26^ In hypertensive populations, rarefaction and remodeling of intramyocardial coronary circulation, along with left ventricular hypertrophy, could contribute to CMD.^27^^,^^28^ Numerous clinical studies have found that hyperlipidemia significantly impacts endothelium-dependent vasomotor function and acts as a major risk factor for CMD, with elevated levels of total cholesterol and low-density lipoprotein cholesterol.^29^^,^^30^

## SNPs associated with CMD

### SNPs associated with coronary microvascular vasoconstriction

**Figure 1:**
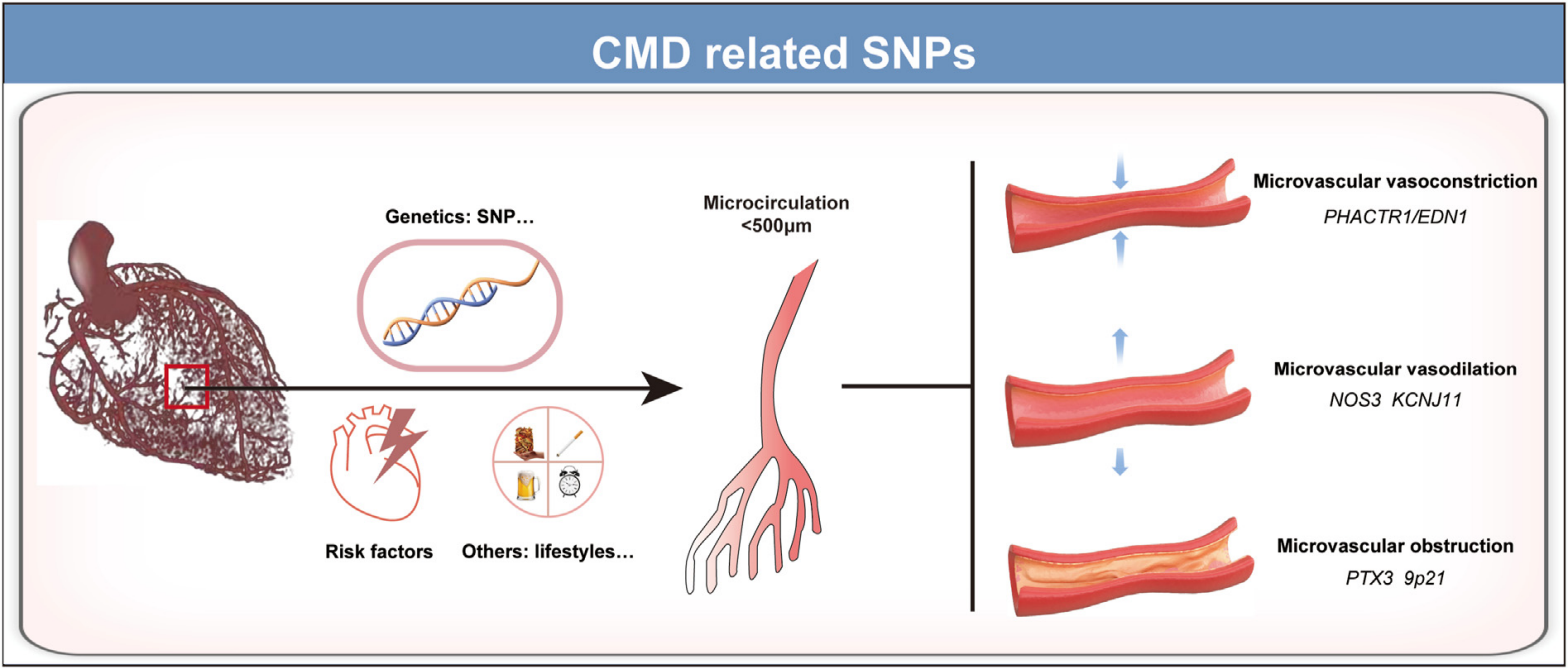
CMD-related SNPs. CMD, coronary microvascular dysfunction; SNP, single nucleotide polymorphism.

The constriction of coronary microvessels has been considered to be the functional mechanism of CMD,^1^ mediated by vasoconstrictors. Increased release of contractile agonists leads to abnormal vasoconstriction. Endothelin-1 (ET-1) induces the construction and remodeling of resistance arteries via the calcium-independent activation of Rho-kinase and the subsequent phosphorylation of the myosin light chain, causing CMD.^21^ The rs9349379-G allele augmented the CMD risk by modulating vasoconstriction with higher plasma ET-1 levels. Patients with the rs9349379-G allele presented peripheral microvessel reactivity to ET-1 and vasoconstriction can be reversed by Zibotentan, an endothelin receptor blocker, indicating a potential targeted intervention for CMD^31^ (Fig. 1 and Table 1).

**Table 1:** CMD associated SNPs.

Target gene(s)	Variant/alleles	Intermediate phenotype	References
PHACTR1/EDN1	rs9349379-G	Plasma ET-1 levels↑	32
NOS3	rs1799983 GT	Endothelial dysfunction	36,37
KCNJ11	rs5215 AA, GA, GG rs5218 CC rs5219 GA, GG rs5216 GG, CC	Kir6.2 subunit of K_ATP+_	37,40
PTX3	rs2305619 AA	Inflammation	41
	rs1333040 TT	Impaired neovessel maturation	42

CMD, coronary microvascular dysfunction; ET-1, Endothelin-1; SNP, single nucleotide polymorphism.

### SNPs associated with coronary microvascular vasodilatation

The diastolic dysfunction of coronary microcirculation includes endothelial-dependent and endothelial-independent vasodilation.^32^ Reduced production and increased degradation of endothelial-derived diastolic factors including NO result in impaired endothelial-dependent vasodilation, while the diastolic disorders of VSMCs lead to damaged endothelial-independent vasodilation.^1^^,^^27^^,^^33^

The production and release of NO are important for endothelial-dependent vasodilation. Synthesized from l-arginine and oxygen by endothelial nitric oxide synthase (eNOS), NO activates the guanylate cyclase pathway or reduces calcium inflow to mediate vasodilation in VSMCs.^34^ Recent studies found that rs1799983 GT, the allelic variant of the eNOS gene *NOS3*, was more represented in both CMD and CAD subjects than controls with normal coronary arteries, revealing that rs1799983_GT is one of the risk factors for CMD and CAD.^35^^,^^36^

Coronary VSMCs mediate the contraction and relaxation of coronary arteries through calcium-dependent signals so that coronary blood flow rapidly adapts to changes in myocardial oxygen supply.^37^ It is suggested that hypoxia-induced coronary vasodilation can be partly explained by hypoxia-induced K_ATP_ _+_ activation.^38^ Researchers reported that the SNPs of *KCNJ11*, encoding for the Kir6.2 subunit of K_ATP+_, play an important role in the susceptibility of CMD and CAD. Rs5215 AA, GA, rs5218 CC, and rs5219 GA were more prevalent in CAD patients, while rs5219 GG increased more in CMD patients.^36^^,^^39^ Additionally, rs5215 GG, rs5216 GG, and rs5216 CC were protective factors for CMD and CAD.^39^

### SNPs associated with coronary microvascular obstruction

CMD may also be secondary to obstruction of the great vessels of the coronary artery after recanalization.^17^ The rs2305619 AA of pentraxin 3 (PTX3), involved in inflammation, was associated with a higher incidence of microvascular obstruction in ST-elevation myocardial infarction patients after primary percutaneous coronary intervention and a higher 30-day mortality.^40^ Furthermore, rs1333040 TT in the 9p21 chromosome was also more represented with microvascular obstruction in such patients after primary percutaneous coronary intervention.^41^

## SNPs associated with CAD for predicting CMD-related SNPs

### SNPs associated with coronary artery vasoconstriction

**Figure 2:**
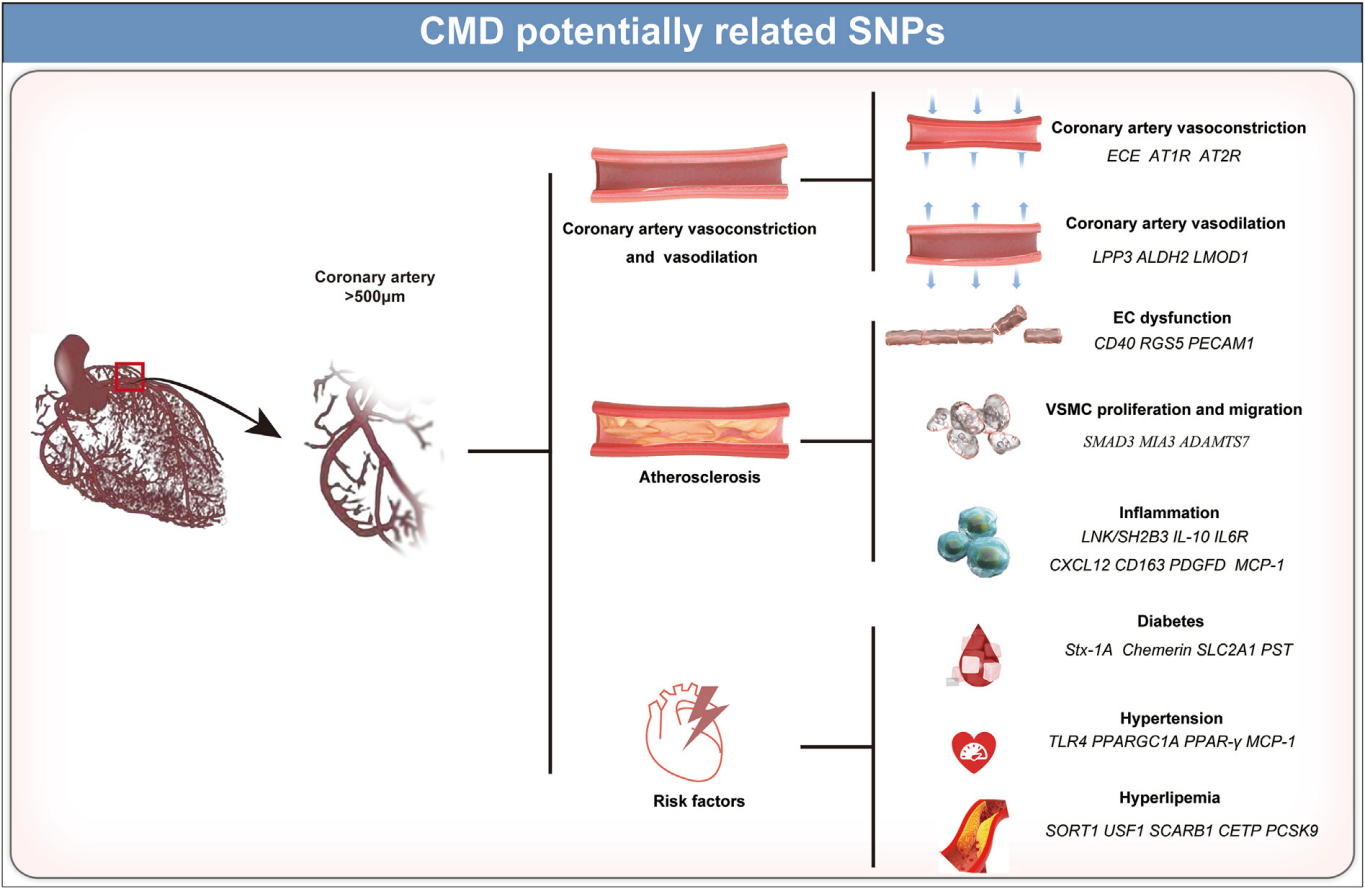
Prediction of CMD-related SNPs. CMD, coronary microvascular dysfunction; EC, endothelial cell; SNP, single nucleotide polymorphism; VSMC, vascular smooth muscle cell.

ET-1-related polymorphisms, which augment ET-1 levels, could increase vascular tone and the subsequent dysfunction of coronary artery vasoconstriction.^42^ Encoded by *EDN1*, the expression of ET-1 is also distally regulated by the *PHACTR1* gene and affected by enzymatic cleavage catalyzed by endothelin-converting enzyme 1. Carriers of at least one copy of the rs6458155C allele of the *EDN1* gene, the minor rs9349379 G allele of the *PHACTR1* gene, and the rs5665 T allele of the *ECE* gene, exhibited increased plasma ET-1 levels and CAD risk.^2^^,^^43^, ^44^, ^45^ Meanwhile, the renin-angiotensin system is also activated to produce excessive angiotensin II, combined with angiotensin II type 1 receptor or type 2 receptor, exerting the vasoconstriction or vasodilation of coronary artery.^46^ The A1166C CC genotype of angiotensin II type 1 receptor was associated with higher CAD risk and higher incidence of sudden cardiac death.^47^ The gene locus of angiotensin II type 2 receptor (−1332 GA) has also been found to impact the occurrence of premature CAD.^48^ Owing to the effect of vascular tone on coronary microvessels, these gene polymorphisms of ET-1, angiotensin II type 1 receptor, and angiotensin II type 2 receptor could complement potential variants associated with CMD (Fig. 2 and Table 2).

**Table 2:** SNPs associated with coronary artery vasoconstriction.

Target gene(s)	Variant/alleles	Intermediate phenotype	References
PHACTR1/EDN1	rs6458155C	Plasma ET-1 levels↑	45
	rs9349379 G		44
ECE	rs5665 T	Endothelin-converting enzyme 1	46
AT1R	A1166C CC	Vasoconstriction	48
AT2R	−1332 GA	Vasoconstriction	49

ET-1, Endothelin-1; SNP, single nucleotide polymorphism.

### SNPs associated with coronary artery vasodilation

ECs mediate coronary artery vasodilation by regulating NO production and the opening and closing of ion channels.^49^ The 894 TT genotype for the *NOS3* gene, located in exon 7, has been reported to contribute to the increased risk of coronary spasm, CAD, and major adverse clinical events including death.^50^ Additionally, the rs3918226C allele was associated with a reduced risk of CAD.^51^ The −786C > T polymorphism, located in the promoter region, was also associated with higher CAD risk.^52^ Reducing the NO-dependent vasodilation, the lipid phosphate phosphatase 3 (*LPP3*) rs17114036 was also associated with the risk of CAD.^53^ It is also reported that the aldehyde dehydrogenase 2 (*ALDH2*) alcohol flushing variant, *ALDH2∗2* (rs671), impacted the risk of CAD by inducing endothelial dysfunction.^54^ Furthermore, coronary microvascular spasm owing to the dysfunction of VSMCs plays an important role in CMD pathogenesis.^55^ The leiomodin 1 (*LMOD1*) gene was implicated in maintaining the phenotype and contractile function of VSMCs.^56^ The T allele of rs2820315, located intronically in the *LMOD1* gene, contributes to a higher CAD risk.^57^ Considering the effects of ECs and VSMCs on coronary microvascular vasodilation, these variations could also be beneficial to predicting the polymorphisms of CMD (Table 3).

**Table 3:** SNPs associated with coronary artery vasodilation.

Target gene(s)	Variant/alleles	Intermediate phenotype	References
NOS3	894 TT rs3918226C	Endothelial dysfunction	51 52
LPP3	rs17114036	Endothelial dysfunction	54
ALDH2	rs671	Endothelial dysfunction	55
LMOD1	rs2820315	VSMC differentiation	58

SNP, single nucleotide polymorphism; VSMC, vascular smooth muscle cell.

## SNPs associated with AS for predicting CMD-related SNPs

AS has been recognized as one of the key factors of CAD pathogenesis, owing to atheromatous narrowing and subsequent occlusion.^58^ A previous study has reported that approximately 80 percent of women with chest pain and no obstructive CAD have AS.^59^ Plaque erosion, fissuring, or rupture induced by AS also leads to the obstruction and increased vasoconstriction of microvessels.^60^ Furthermore, focal or diffuse CA has also been suggested to be associated with CMD development.^18^ EC dysfunction, changeable VSMC proliferation and migration, and inflammation, could be potential genetic links between AS and CMD.^60^, ^61^, ^62^ Therefore, the SNPs associated with AS have great implications for discovering novel variants of CMD (Table 4).

**Table 4:** SNPs associated with coronary atherosclerosis.

Target gene(s)	Variant/alleles	Intermediate phenotype	References
CD40	rs1883832C	Endothelial dysfunction	65
RGS5	rs1056515	Endothelial dysfunction	66
SMAD3	rs17293632 T	VSMC proliferation	67
	rs41291957 G > A	VSMC phenotypic switch	71
ADAMTS7	rs3825807 rs1994016	VSMC migration	73 74
PECAM1	rs1867624	Vascular barrier integrity and inflammation	58
MIA3	rs67180937 G	lower VSMC proliferation and harmful phenotypic transitions	69
LNK/SH2B3	R262W	Inflammation	75
IL-10	−592A/C	Inflammation	76
IL6R	rs2228145 rs4537545 rs7529229	Inflammation	77
CXCL12	rs1746048	Inflammation	78,79
CD163	rs7136716	Inflammation	80
PDGFD	rs974819	Inflammation	82
MCP-1	rs2857656 CC	Inflammation	83

SNP, single nucleotide polymorphism; VSMC, vascular smooth muscle cell.

### SNPs associated with EC dysfunction in AS

EC dysfunction is pivotal to the initiation and progression of AS.^62^ CD40 is involved in the activation of ECs and adhesion of leucocytes, and its interaction with CD40 ligand plays an important role in AS.^63^ A previous study revealed that the rs1883832C allele increased the risk of CAD through enhancing CD40 expression and subsequent monocyte adhesion.^64^ Moreover, the rs1056515 variant of regulator of G protein signaling 5 (*RGS5*), accounting for the decreased gene expression, was associated with impaired EC function and increased AS risk.^65^ Considering the role EC dysfunction plays in both CMD and AS, these SNPs associated with EC dysfunction might also be implicated in CMD development.

### SNPs associated with VSMC proliferation and migration in AS

Genome-wide association studies (GWASs)have reported that the rs17293632 T allele of SMAD family member 3 (*SMAD3*) was associated with reduced SMAD3 expression, inhibiting VSMC proliferation and protecting against CAD.^66^^,^^67^ Similarly, the rs67180937 G allele of MIA3 was associated with lower VSMC proliferation and harmful phenotypic transitions in AS.^68^ MiR-143 and miR-145 in VSMC could regulate the proliferation of VSMC, and be associated with AS.^69^ The rs41291957 G > A variant has been reported to affect miR-143 and miR-145 expression to facilitate VSMC switch to differentiated/contractile phenotype, contributing to a lower CAD risk.^70^ A disintegrin and metalloproteinase with thrombospondin 7 (ADAMTS7) has been found to promote VSMC migration by degrading extracellular matrix.^71^ The rs3825807 variant has been found to modulate ADAMTS7 maturation to protect against CAD, and rs1994016 of the *ADAMTS7* gene was associated with increased risks of CAD and AS.^72^^,^^73^ Given that VSMC proliferation and migration affect CMD, these loci have great potential for predicting CMD-related SNPs.

### SNPs associated with inflammation in AS

Platelet endothelial cell adhesion molecule-1 (PECAM1) mediates the protection of vascular barrier integrity, the disruption of which leads to the development of chronic inflammatory diseases such as AS.^57^ The rs1867624 variant reduced PECAM1 expression, destroyed coronary barriers, and increased CAD risk.^57^ The variant of *LNK/SH2B3* R262W, affecting platelet–neutrophil aggregates, also displayed increased CAD risk in individuals with JAK2^VF^ mutation.^74^ Furthermore, the −592A/C polymorphism of anti-inflammatory factor interleukin-10 (*IL-10*) was associated with slow coronary flow and AS in the Han Chinese population.^75^ Minor alleles of rs2228145, rs4537545, and rs7529229 of the interleukin 6 receptor (*IL6R*) gene have been also reported to be negatively associated with CAD risk.^76^ The rs1746048 variant of C-X-C motif chemokine ligand 12 (*CXCL12*) modulating plasma CXCL12 levels, was associated with CAD risk and related complications.^77^^,^^78^ Though the intake of hemoglobin by CD163 inducing a pathogenic or protective macrophage phenotype in AS remains controversial, the minor allele of the rs7136716 genotype could mediate microvessel density and impact the risks of CAD and myocardial infarction by regulating CD163 expression.^79^ A previous study reported that platelet-derived growth factor-D facilitated matrix metalloproteinase activity and monocyte migration in AS.^80^ In the Han Chinese population, the SNP rs974819 of the PDGFD gene was sex-dependent and influenced CAD risk.^81^ Besides, monocyte chemoattractant protein 1 (MCP-1) could promote recruitment of macrophages into atherosclerotic plaque. The rs2857656 CC genotype of MCP-1 contributed to a higher prevalence of carotid artery plaque.^82^ Since inflammation is one of the important pathogenic mechanisms of CMD, these variants might be also associated with CMD risk or complications.

## SNPs associated with CMD risk factors for predicting CMD-related SNPs

### Diabetes-associated SNPs

Diabetes leads to endothelial dysfunction, changes in the levels of hormones, and alteration in the metabolism of VSMCs, which in turn cause the development of microvascular abnormalities.^83^ During chronic diabetes, hyperglycemia and insulin resistance reduce eNOS expression in ECs, which causes decreased NO production, decreased endothelium-dependent relaxation, and CMD.^84^ Functionally impairment of VSMCs in diabetes also aggravated macrovascular complications such as CAD^85^.

The soluble NSF attachment protein receptor (SNARE) complex was involved in metabolic diseases.^86^ The rs4717806 A and rs2293489 T minor alleles of syntaxin 1A (*Stx-1A*), a protein component of the SNARE complex, were associated with CAD risk.^87^ Previous studies have reported chemerin-induced vascular inflammation and endothelial dysfunction.^88^ Chemerin rs17173608 has been found to be a promising indicator for predicting insulin resistance and assessing the severity of CAD.^89^ Polymorphisms of solute carrier family 2 facilitated glucose transporter member 1 (*SLC2A1*) was associated with diabetic microangiopathy, possibly due to their role in the proliferation and extracellular matrix synthesis of VSMCs.^90^ The rs1385129 of *SLC2A1* was associated with the prevalence of cardiovascular complications in diabetic patients.^91^ The rs9658664 of pancreastatin (*PST*), the peptide of which regulates glucose/insulin homeostasis, has conferred an increased risk for diabetes, hypertension, and CAD.^92^ As one of the risk factors, diabetes-related SNPs affecting coronary artery structure and function could enlighten us in SNP prediction in CMD (Table 5).

**Table 5:** SNPs associated with CMD risk factors.

Target gene(s)	Variant/alleles	Intermediate phenotype	References
Stx-1A	rs2293489 T rs4717806 A	Metabolic syndrome and insulin resistance	88
Chemerin	rs17173608	Vascular inflammation and endothelial dysfunction	90
SLC2A1	rs1385129	Proliferation and extracellular matrix synthesis of VSMCs	92
PST	rs9658664	Diabetes	93
SORT1	rs599839	LDL-C level↓	97
USF1	rs11576837	Hyperlipidemia	98
SCARB1	rs5888	HDL↑	100
CETP	rs1800775	HDL	101
PCSK9	rs11206510 rs11591147	LDL↓	103 104
AGT	M235T	Coronary artery calcium	106,107
TLR4	896 G	Blood pressure and pulse pressure↓	108
PPARGC1A	Gly482Ser	Hypertension	109
PPAR-γ	rs1801282	Glucose, cholesterol, triglyceride and ALT↑	110
MCP-1	2518 A/G	Blood pressure↑	111

CMD, coronary microvascular dysfunction; HDL, high density lipoprotein; LDL, low density lipoprotein; SNP, single nucleotide polymorphism; VSMC, vascular smooth muscle cell.

### Hyperlipidemia-associated SNPs

Hyperlipidemia has been recognized to play an important role in both CAD and CMD.^33^^,^^93^ Lowering lipoprotein levels has been reported to improve CMD in hyperlipidemic patients.^94^ Previous research highlighted that the association of the rs599839 G-allele of *SORT1* with reduced low-density lipoprotein and triglyceride levels, and observed the decreased prevalence of CAD and myocardial infarction in subjects with the rs599839 GG genotype.^95^ Upstream stimulatory factor 1 (USF1) is a transcription factor associated with familial combined hyperlipidemia and CAD. The rs11576837 variant reduces *USF1* expression, improves insulin sensitivity and lipid profiles, and alleviates AS.^96^ Scavenger receptor B1, encoded by the *SCARB1* gene, mediates selective uptake of high-density lipoprotein cholesteryl esters into steroidogenic cells and the liver, impacting the development of AS through apolipoprotein B-containing particles.^97^ It has been shown that the *SCARB1* rs5888 AA genotype represents a higher level of large-sized high-density lipoprotein subtype, whereas the population with rs5888 GA and GG types shows increased CAD risk.^98^ The rs1800775 variant, located in the promoter of the cholesteryl ester transfer protein (CETP) gene, was also associated with plasma high-density lipoprotein cholesterol level and CAD risk.^99^ Proprotein convertase subtilisin/kexin type-9 (PCSK9), binding to low-density lipoprotein receptors on the cell surface and participating in lysosomal degradation, could be a target for dyslipidemia.^100^ The genetic variants rs11206510 and rs11591147 were associated with cholesterol levels and contributed to a lower risk of myocardial infarction or CAD.^101^^,^^102^ These hyperlipidemia-associated loci could be used to predict the potential risk SNPs of CMD.

### Hypertension-associated SNPs

Hypertension has been recognized as one of the most important components among genetic risk factors of CAD.^103^ The angiotensinogen (*AGT*) gene M235T variant was linked with CAD risk and coronary artery calcium in the CAD population.^104^^,^^105^ A previous study reported that CAD patients with the Toll-like receptor 4 *(TLR4)* 896 G allele had lower systolic blood pressure and pulse pressure, compared with TLR4 896 A/A allele carrier.^106^ The Gly482Ser variant of peroxisome proliferator-activated receptor-gamma coactivator 1-alpha (*PPARGC1A*), a gene related to energy metabolism and mitochondrial biogenesis, was associated with hypertension and CAD.^107^ In addition, the loci of several genes including *CYP17A1*, *GUY1A1*, and *ARHGAP42* were also found to be associated with hypertension and CAD.^103^ Hypertension could be a risk for peroxisome proliferator-activated receptor-gamma (*PPAR*-γ) rs1801282 mutation in CAD subjects.^108^ The SNP of MCP-1 2518 A/G was also linked with blood pressure in asymptomatic patients with ischemic heart disease.^109^ These hypertension-associated loci could also benefit our prediction of CMD risk SNPs.

## Conclusion

CMD refers to the structural and functional remodeling of the coronary microcirculation, and has a significant impact on the prognosis of concomitant diseases such as CAD.^1^^,^^110^ Accordingly, CMD has become increasingly crucial to the diagnosis and treatment of coronary heart disease.^111^ Currently, several obstacles exist in ensuring successful the prevention and treatment of CMD, including unclear pathogenic mechanisms, cumbersome diagnostic procedures, and lacking targeted interventions. Promisingly, it is particularly necessary to explore the pathogenesis and targeted intervention of CMD. A large number of epidemiological studies and GWAS have revealed that SNPs play an important role in the occurrence and development of a variety of cardiovascular diseases, and investigation of these SNPs seems to promise new insights into CMD pathogenesis and potential treatments. Consistently, predicting and screening CMD associated SNPs not only contributes to the early diagnosis of CMD-susceptible populations but also provides the possibility of targeted intervention for CMD. However, few studies have identified SNPs associated with CMD. Most extant studies focused on CMD pathogenesis such as coronary systolic function, diastolic function, and coronary microvascular obstruction, and some of which have been found to have a promising clinical application. To further explore more SNPs with a strong correlation with CMD risk, our review also illustrates potential CMD risk variants from cardiovascular diseases with similar mechanisms and risk factors. These loci could benefit the investigation of CMD-related SNPs and offer targeted interventions to be developed in the future.

## Author contributions

Z.H.Z. and F.D. conceived and designed the project; D.Y.T., J.L., Z.H.Z., and F.D. wrote the manuscript; D.Y.T., J.L., and Q.Y.Y. drew the figures and tables; X.Y.L. checked for spelling mistakes.

## Conflict of interests

These authors declared no conflict of interests.

## Funding

This work was supported by the Chongqing Key Project of Science and Technology Joint Medical Research (China) (No. 2019ZDXM013 to Z.H.Z.) and the Chongqing Talent Program (China) (No. CQYC20210303360 to Z.H.Z.).
